# Clinical case: Combined pulmonary fibrosis and emphysema with pulmonary hypertension – clinical management

**DOI:** 10.1186/1756-0500-6-S1-S2

**Published:** 2013-04-16

**Authors:** Vincent Cottin

**Affiliations:** 1 Hospices Civils de Lyon, Hôpital Louis Pradel, Service de pneumologie – Centre de référence national des maladies pulmonaires rares, Université Claude Bernard Lyon, Lyon, France

## Abstract

**Background:**

Combined idiopathic pulmonary fibrosis (IPF) with pulmonary emphysema (CPFE) is a syndrome with a characteristic presentation of upper lobe emphysema and lower lobe fibrosis. While CPFE is a strong determinant of secondary precapillary pulmonary hypertension (PH), there is limited evidence regarding the management of patients with CPFE and PH.

**Case presentation:**

A 63 year-old male presented in 2006 with dyspnoea on exertion having quit smoking in 2003. Clinical examination, together with high resolution computed tomography, bronchoalveolar lavage, and echocardiographic assessments, suggested a diagnosis of CPFE without PH. In 2007, the patient received intravenous cyclophosphamide, N-acetylcysteine, and short-term anticoagulation treatment. Due to remission of acute exacerbations, the patient received triple combination therapy (prednisone, N-acetylcysteine and azathioprine). Upon progressive clinical worsening, long-term supplemental oxygen therapy was initiated in 2009. Repeated right heart catheterisation in 2011 confirmed PH and worsening pulmonary haemodynamics, and off-label ambrisentan therapy was initiated. Dyspnoea remained at follow-up, although significant haemodynamic improvement was observed.

**Conclusion:**

CFPE is a distinct but under-recognized and common syndrome with a characteristic presentation. Further studies are needed to ascertain the etiology, morbidity, and mortality of CPEF with or without PH, and to evaluate novel management options.

## Introduction

There is an increasing awareness of comorbid conditions frequently associated with idiopathic pulmonary fibrosis (IPF), including emphysema, cardiovascular disease, thromboembolic disease, and obstructive sleep apnoea. Recent retrospective data suggest that 21 to 33% of patients with IPF may have co-existing emphysema [1-3]. The association of emphysema with IPF has been termed the combined pulmonary fibrosis and emphysema (CPFE) syndrome [4,5] to account for the characteristic clinical, functional, imaging, and outcome features. Characteristics of patients with CPFE syndrome include male predominance, tobacco smoking, severe dyspnoea, subnormal spirometry findings, severely impaired transfer capacity for carbon monoxide [4,6,7], hypoxemia upon exercise, high frequency of paraseptal emphysema, and a high probability (30–50%) of severe pulmonary hypertension (PH) impacting upon an already poor prognosis [2,4]. However, very limited evidence is available regarding the management of patients with PH in the CPFE syndrome.

## Clinical case

### Observation

A 63 year-old man presented in 2006 with dyspnoea on exertion (New York Heart Association (NYHA) functional class II). He had smoked 20 pack-years and quit smoking in 2003, had no professional exposure, and had a history of hypercholesterolemia treated with atorvastatin, and surgery for prostatic adenocarcinoma followed by complete remission. Clinical examination demonstrated fine ’Velcro‘-like crackles of the lung bases, with no finger clubbing, and the absence of clinical signs of connective tissue disease. The patient’s body mass index was 25 kg/m².

### Diagnostic tests

**Figure 1:**
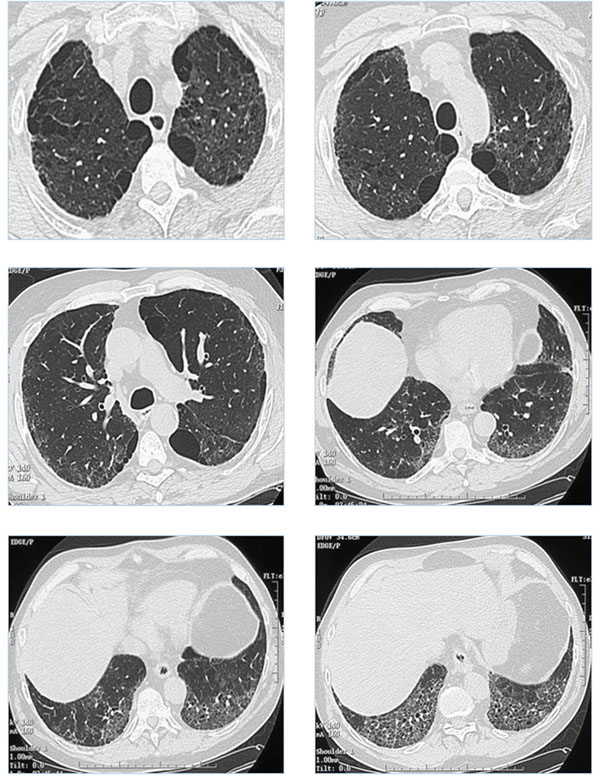
High resolution computed tomography (HRCT) of the chest demonstrating predominantly paraseptal emphysema of the upper areas of the lungs, and interstitial lung disease of the basal regions, with intralobular reticulation and ground glass opacities.

IPF was suspected, however lung volumes were subnormal, with forced vital capacity (FVC) of 91% of predicted, total lung capacity (TLC) of 79%, and a residual volume of 64%. Forced expiratory volume in one second (FEV_1_) was 85% of predicted; the FEV_1_/FVC was 71%. Transfer capacity for carbon monoxide (DLco) was 72% and transfer coefficient was 81%. Partial arterial pressure of oxygen was 11.7 kPa, decreasing to 8.2 kPa with exercise. High resolution computed tomography (HRCT) of the chest demonstrated predominantly paraseptal emphysema of the upper zones of the lungs, and interstitial lung disease of the basal regions, with intralobular reticulation and ground glass opacities (Figure 1). Bronchoalveolar lavage (BAL) differential cell counts showed 10% neutrophils, 1% eosinophils, 2% lymphocytes, and 87% macrophages. Echocardiography did not demonstrate PH. The patient was diagnosed with CPFE syndrome.

### Treatment and patient management

In 2007, the patient presented with acute exacerbation of pulmonary fibrosis fulfilling international criteria [8], and BAL culture was negative; pulmonary embolism and left heart disease were excluded. The patient received intravenous cyclophosphamide (6-monthly pulses), N-acetylcysteine, and short-term anticoagulation treatment, but experienced clinical and HRCT remission of the acute exacerbation. He was further treated with triple combination therapy (prednisone, N-acetylcysteine 1.8 g per day, and azathioprine), with progressive clinical worsening despite therapy.

In 2009, dyspnoea was NYHA functional class III. FVC was 67% of predicted, TLC was 52%, residual volume 33%, FEV_1_ 66%, FEV_1_/FVC 75%, DLco 21% and Kco 50%. Long-term supplemental oxygen therapy was initiated, and PaO_2_ under 2 L/min supplemental oxygen was 11.2 kPa. The 6-min walk distance was 240 m, with peripheral oxygen saturation of 76% while breathing 4 L/min supplemental oxygen. The patient presented with episodes of syncope at exertion. Echocardiography demonstrated dilated right heart cavities, with estimated systolic pulmonary arterial pressure of 45 mmHg. Right heart catheterisation confirmed precapillary PH (Table 1).

**Table 1:** Right heart catheterisation in the patient with combined pulmonary fibrosis and emphysema.

Date	May 2009	Feb 2011	June 2011
PAP m (mmHg)	26	38	25
PCWP (mmHg)	8	12	9
SvO2 (%)	68	59	-
Cardiac index (L/min/m²)	2.25	3.39	3.02
PVR (dyn/s/cm^5^)	356	488	246
RAP (mmHg)	8	7	5
Vasoreactivity	Negative	-	-
Treatment	None	Initiation of ambrisentan	ambrisentan

In 2011, repeated catheterisation showed worsening of pulmonary haemodynamics, and off-label ambrisentan therapy (5 mg per day) was initiated, with no change in oxygen saturation and PaO_2_. At follow-up evaluation, dyspnoea was unchanged; however significant haemodynamic improvement was observed (Table 1), and 6-min walk distance improved by 27 m.

## Discussion

This patient case highlights a number of characteristics of the CPFE syndrome that contribute to distinctly different management compared with IPF alone (i.e. in the absence of emphysema). CPFE is a *syndrome* with characteristic presentation, including very low diffusion capacity contrasting with subnormal spirometry, occurring in heavy smokers with severe dyspnoea and exercise limitation. It may be overlooked due to subnormal lung volumes, however gas exchanges are severely altered. Despite moderate or severe emphysema, a large proportion of patients with CPFE have FEV_1_/FVC >70% indicating that GOLD criteria for chronic obstructive lung disease may not be applicable [3]. In addition, diagnostic criteria of IPF may not apply, owing to difficulties in ascertaining honeycomb changes in patients with associated emphysema UIP [9] and a high frequency of mild-to-moderate ground glass opacities [4].

A limited amount of data is available regarding lung pathology in patients with CPFE. Indeed, severe alteration of gas exchange and emphysema features at imaging may explain why lung biopsy is rarely performed, as in this presented case. A variety of pathology patterns of pulmonary fibrosis have been reported in patients with CPFE, including predominantly UIP pattern [10], however nonspecific interstitial pneumonia [11], desquamative interstitial pneumonia (with extensive fibrosis), respiratory bronchiolitis, associated interstitial lung disease, airspace enlargement with fibrosis, or unclassifiable smoking-related interstitial fibrosis, may be observed.

This case further indicates that the natural course of disease in CPFE may encompass episodes of acute exacerbation of pulmonary fibrosis, a complication seldom reported previously in CPFE. Further, the presence of significant emphysema impacts FVC measurement, and thus changes in FVC alone may not be a reliable indicator of disease [7,12]. Patients with CPFE should be excluded from IPF clinical trials [5]. In fact, a decline in FEV_1_ by 10% or more at 6 or 12 months may be useful in assessing disease progression [3], which contrasts with the monitoring of IPF alone using serial changes in FVC and DLco. The main predictor of subsequent mortality is precapillary pulmonary hypertension [2,4], which portends a dismal prognosis [13]. Whether survival of patients with IPF is impacted by coexistent emphysema (e.g. CPFE) is controversial, due to difficulties in controlling for disease severity [2,14]. A composite physiologic index may account for the extent of disease [15].

There are currently no specific recommendations for the treatment of pulmonary fibrosis, emphysema or PH in the setting of CPFE. It is unknown whether treating these components of disease influences clinical outcomes. Patients with CPFE are likely to require long-term oxygen therapy. The presented case indicates that therapy specific for pulmonary hypertension may improve haemodynamics, as in other isolated reports [13,16], but the potential clinical and survival benefit is unknown. Results of a recent trial (‘ARTEMIS-IPF‘, NCT00768300) have showed that ambrisentan therapy is not beneficial and may even be deleterious in patients with IPF, regardless of associated PH, and should now be avoided in this condition. Treatment specific for PH may in some cases impair oxygenation by worsening ventilation/perfusion mismatch. Recent data have recommended against combination therapy with prednisone, azathioprine, and high-dose N-acetylcysteine in IPF management [17]. Monotherapy using N-acetylcysteine is currently widely used in this setting, yet without evidence.

In conclusion, CPFE is a *syndrome* with characteristic presentation, and with a prognosis dominated by precapillary PH-associated with a poor prognosis, despite moderate haemodynamic severity. Further study is required regarding therapies targeting pulmonary fibrosis or PH.

## Competing interest

Vincent Cottin has received fees for speaking from Intermune, Boehringer Ingelheim, GSK, and Actelion, and has participated as a member of steering committees, a member of data safety monitoring boards or as an investigator to clinical trials sponsored by Actelion, Boehringer Ingelheim, Gilead, and Intermune Inc.

## Patient consent

Written informed consent was obtained from the patient for publication of this case report and any accompanying images. A copy of the written consent is available for review by the Editor of this journal.

## Declarations

This article has been published as part of *BMC Research Notes* Volume 6 Supplement 1, 2013:IPF in 2011 – Key updates on guidelines and therapeutics. The full contents of the supplement are available online at http://www.biomedcentral.com/bmcresnotes/supplements/6/S1. This supplement originates from presentations given at the symposium “AIR: Advancing IPF Research. Working together to translate IPF research into practice” held in Berlin in November 2011. The publication was supported by InterMed Communications with funding from InterMune, AG. The content was proposed by InterMed Communications and developed with the journal. All articles in the supplement have undergone the journal’s standard peer review process.
